# Induced-proximity therapeutics for targeted protein and RNA degradation: An organic chemistry Perspective-A review

**DOI:** 10.1016/j.crstbi.2026.100181

**Published:** 2026-01-31

**Authors:** Mohammad Rizehbandi, Ehsan Dadfar, Mahyar Rezaei Nami, Mahdi Rezaei Nami, Mehran Rezaei Nami

**Affiliations:** a Department of Organic Chemistry, Faculty of Chemistry, University of Guilan, Rasht, Iran; b Chemistry Department, Graduate Faculty, Islamic Azad University, Arak Branch, P. O. Box 38135-567, Arak, Iran; c Department of Organic and Polymer Chemistry, Faculty of Chemistry, Kharazmi University, P. O. Box: 15719-14911, Tehran, Iran; d Department of Organic Chemistry, Shahid Beheshti University, 1983969411, Tehran, Iran; e Department of Chemistry, Science and Research Branch, Islamic Azad University, Tehran, Iran

**Keywords:** Targeted protein degradation, PROTACs, Molecular glues, Linker chemistry, Structure–activity relationships (SAR), RiboTACs

## Abstract

Induced-proximity therapeutics have emerged as a transformative paradigm in chemical biology and drug discovery, enabling selective control of cellular processes beyond conventional inhibitors. Between 2020 and 2025, major progress has been achieved across five modalities: proteolysis-targeting chimeras (PROTACs), molecular glues, lysosome-targeting chimeras (LYTACs), autophagy-targeting chimeras (AUTACs) and related tethering strategies, and ribonuclease-targeting chimeras (RIBOTACs). Each exploits endogenous degradation or regulatory pathways using chemically engineered bifunctional or monofunctional small molecules, thereby expanding the druggable proteome and transcriptome. This review provides a comparative analysis of their underlying organic chemistry, design principles, and mechanistic diversity. We highlight structure activity relationships, linker optimization, and chemical motifs that govern induced proximity and degradation efficiency. Advances in ligand discovery, modular synthetic methodologies, and strategies to improve pharmacokinetics and tissue selectivity are emphasized. Schematic diagrams illustrate key mechanistic steps, offering a visual framework for comparing similarities and differences across approaches. While prior reviews have focused on mechanistic and pharmacological aspects, our perspective emphasizes synthetic strategies, linker chemistry, SAR studies, and ligand optimization principles that underpin each degrader class. We examine how advances in synthetic design, modular assembly, and chemical reprogramming of ligases or receptors have broadened therapeutic potential. By critically assessing strengths, limitations, and chemical challenges across modalities, we propose a unifying organic chemistry perspective that distinguishes induced-proximity strategies from conventional small-molecule inhibition and outlines future opportunities in degrader design.

## Highlights

• Degrader modalities—including PROTACs, molecular glues, LYTACs, AUTACs/ATTECs, RIBOTACs, and emerging platforms such as DUBTACs and TransTACs—use induced proximity to modulate proteins, organelles, and RNA target.
• Organic-chemistry design challenges, such as linker installation, stereochemical control, glycan conjugation, and RNA-binder optimization, critically shape degrader function and developability.
• Linker class (rigid, flexible, cleavable, macrocyclic, polarity-masking) strongly influences ternary-complex formation, degradation efficiency, and pharmacokinetic behavior.

## Graphical abstract

**Figure:**
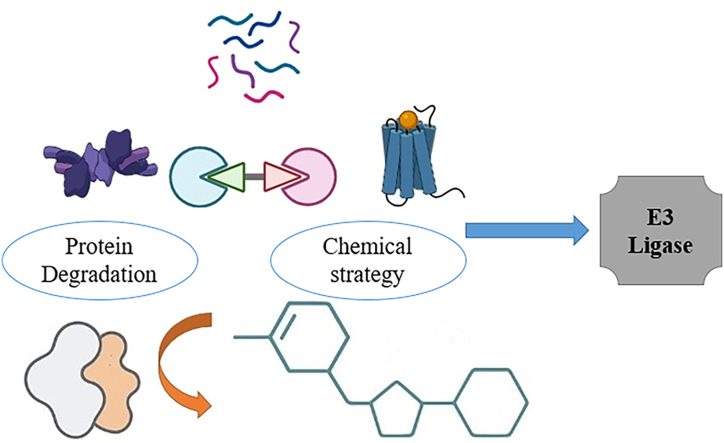

## 1 Introduction

Induced-proximity therapeutics have emerged as a powerful modality in drug discovery, enabling the selective elimination of disease-causing biomolecules by harnessing endogenous degradation pathways. Unlike traditional inhibitors that require sustained high-affinity binding, these agents act catalytically: a single small molecule can trigger the destruction of multiple target copies, thereby expanding the scope of “druggable” targets beyond what was historically feasible (Békés et al., 2022; Alabi and Crews, 2021). The concept was first exemplified about two decades ago with proteolysis-targeting chimeras (PROTACs), and by 2020 clinical proof-of-concept had been established for PROTAC drugs against oncology targets (Alabi and Crews, 2021). Since then, the field has rapidly diversified, giving rise to a broad arsenal of induced-proximity strategies. These include PROTACs and molecular glue degraders that exploit the ubiquitin–proteasome system, lysosome-directed approaches such as LYTACs (lysosome-targeting chimeras), and autophagy-based tactics (e.g., AUTACs, ATTECs, AUTOTACs). More recently, induced proximity has been extended to RNA with RIBOTACs (ribonuclease-targeting chimeras) that promote degradation of disease-relevant transcripts. In all cases, carefully designed organic molecules or conjugates bring a target into proximity with a cellular “effector” (E3 ligases, hydrolases, or receptors) to initiate its elimination. This review focuses on the organic chemistry and mechanisms underlying these induced-proximity modalities, highlighting key advances from 2020 to 2025 and their implications for drug development. From a synthetic chemistry perspective, induced-proximity therapeutics are defined as much by their chemical architecture as by their biological mechanism. Ligand discovery, modular linker strategies, and systematic structure–activity relationship (SAR) studies are core organic chemistry principles that have enabled targeted protein and RNA degradation. Linker length, polarity, and rigidity are tuned using well-established synthetic transformations such as amide bond formation, click chemistry, and cross-coupling reactions, while fragment-based ligand design and late-stage functionalization guide the optimization of warheads. Throughout this review, we integrate mechanistic insight with the underlying organic chemistry that governs synthetic feasibility, molecular stability, and degradation efficiency.

## 2 Induced-proximity modalities

### 2.1 PROTACs: heterobifunctional degraders via the ubiquitin–proteasome pathway

Proteolysis-targeting chimeras PROTACs induce ubiquitination by bringing the POI into proximity with an E3 ligase (for a detailed description, see Section 2.1). By forming a ternary complex between the POI and the E3 ligase, PROTACs promote ubiquitination of the target and its subsequent degradation by the proteasome (Békés et al., 2022; Alabi and Crews, 2021). This catalytic mechanism allows a single PROTAC molecule to induce multiple rounds of target depletion (Baisden et al., 2021). PROTAC design therefore hinges on optimizing three elements: the POI ligand, the E3 ligase ligand, and the linker connecting them.

Early PROTACs relied on peptide-based recognition motifs, but contemporary designs employ compact small-molecule binders discovered through medicinal chemistry or fragment-based approaches (Banik et al., 2020; Ahn et al., 2021). For example, the widely used VHL-recruiting ligand was derived by mimicking the hydroxyproline residue of HIF-1α and appending linkers at positions informed by co-crystal structures (Banik et al., 2020; Ahn et al., 2021; Arimoto et al., 2021). Thalidomide analogues such as pomalidomide, which bind cereblon (CRBN), emerged in the 2010s as another versatile class of E3-recruiting warheads (Ahn et al., 2021). As a result, VHL- and CRBN-based PROTACs dominate current designs, although other ligases including MDM2 and IAP have also been exploited (Li et al., 2019a). Recent work has further expanded the E3 repertoire: covalent ligands for RNF4 (Ji et al., 2022), DCAF16 (Haj-Yahia et al., 2023a), KEAP1, RNF114, and additional ligases have been identified and incorporated into PROTAC architectures, enabling degradation of targets in specific subcellular compartments or tissues (Meyer et al., 2022; Cornu et al., 2025). For instance, a covalent DCAF16 binder linked to JQ1 (a bromodomain inhibitor) yielded a PROTAC that achieved nucleus-selective degradation of BRD4 (Haj-Yahia et al., 2023a; Meyer et al., 2022; Cornu et al., 2025; Kim et al., 2024), while the natural product nimbolide, which recruits RNF114, has been used to degrade oncogenic proteins (Cornu et al., 2025). These examples illustrate how advances in synthetic chemistry and covalent warhead design are expanding the scope of PROTACs by accessing new E3 ligases and binding modes.

**Fig. 1:**
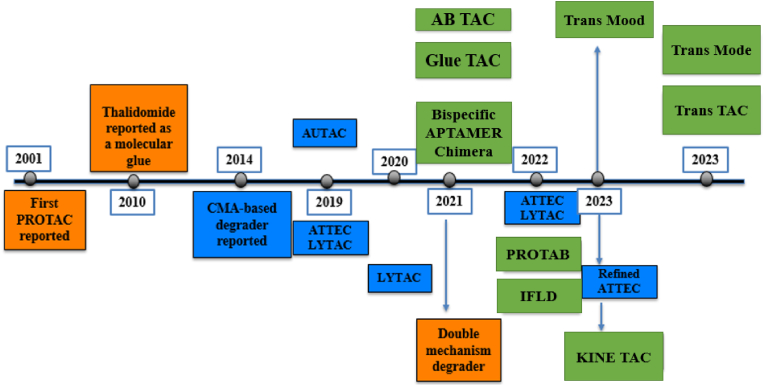
Historical Timeline of Induced-Proximity Degrader Technologies (Modified and redrawn by the authors).

**Fig. 2:**
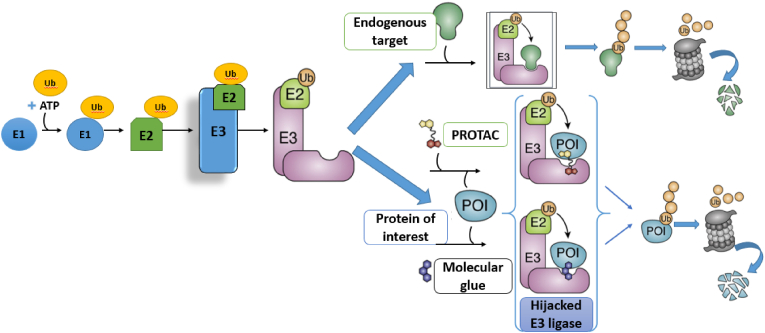
Mechanism of PROTACs vs. Molecular Glue Degraders (UPS Hijacking). Upper path: In the normal ubiquitin–proteasome system, an E3 ligase brings a substrate protein together with an E2 enzyme, catalyzing ubiquitination of the substrate. Polyubiquitinated proteins are then recognized and degraded by the proteasome (Alabi and Crews, 2021).[2] (Modified and redrawn by the authors).

The construction of such heterobifunctional molecules relies on robust and modular organic transformations. Typical PROTAC syntheses use amide formation, esterification, and carbamate linkages, combined with orthogonal protecting-group strategies, to assemble E3 ligase ligands, linkers, and target binders in a controlled manner. More recent approaches employ Cu(I)-catalyzed azide–alkyne cycloaddition (“click chemistry”) to introduce PEG or alkyl spacers efficiently, and prodrug-like linkers that contain cleavable ester or carbonate motifs to modulate pharmacokinetics. Medicinal chemistry optimization of PROTACs involves iterative SAR cycles, in which subtle modifications such as replacing a methylene unit with an ether, altering linker length, or shifting a linker attachment site can profoundly influence ternary complex stability, cellular permeability, and degradation kinetics. Between 2020 and 2025, PROTACs have experienced rapid growth in both academia and industry. More than twenty candidates have advanced into clinical trials for indications including cancer, with targets such as the androgen receptor, estrogen receptor, BTK, and IRAK4 (Qin et al., 2022). Notably, Arvinas’ ARV-110 and ARV-471 have demonstrated target degradation in patients, providing clinical validation of the PROTAC concept in oncology. The catalytic mode of action of PROTACs can offer advantages over classical inhibitors: degradation can overcome certain resistance mutations (as long as the ligand still binds the target) (Konstantinidou et al., 2024), abolish both catalytic and scaffolding functions, and sustain functional knockdown until new protein is synthesized. Consequently, PROTACs may allow less frequent dosing while maintaining deep target suppression, an especially attractive feature in cancer therapy (Zheng et al., 2024). The general principles of induced-proximity therapeutics are summarized in Fig. 1, which illustrates how heterobifunctional and monovalent small molecules can direct degradation or stabilization pathways by chemically bridging a target biomolecule and an effector protein. This schematic emphasizes the unifying concept that underlies all modalities discussed in this review: ligand discovery, linker engineering, and SAR-driven optimization are central organic chemistry tools that enable diverse yet mechanistically related biological outcomes (see Fig. 2).

Key milestones in the development of targeted degradation strategies are shown on a time line (Békés et al., 2022). Early breakthroughs include the first PROTAC in 2001 (orange, representing ubiquitin–proteasome system [UPS]-based degraders) and the recognition of thalidomide as a molecular glue in 2010 (orange) (Békés et al., 2022). Subsequent innovations (2014–2024) introduced lysosome-directed and autophagy-based approaches (blue shades). Light-blue entries mark autophagy–lysosomal pathway degraders (e.g. CMA-based degrader, AUTAC in 2019, ATTEC in 2020, AUTOTAC in 2022), while dark-blue entries mark endosome–lysosome pathway degraders (e.g. LYTAC in 2020, AbTACs/GlueTACs, bispecific aptamer chimeras, TransTAC/TransMoDE in 2023–2024) (Békés et al., 2022). This timeline highlights the rapid expansion of induced-proximity therapeutics from the initial PROTAC concept to a diverse “toolbox” of strategies by 2025.

From a chemistry perspective, PROTAC design and optimization – sometimes dubbed “linkerology” is a complex multidimensional challenge (Takahashi et al., 2023; Zhang et al., 2025). The length, composition, and attachment points of the linker profoundly influence the induced proximity and ubiquitination efficiency of the target. For example, a series of “HaloPROTAC” degraders was synthesized with varying polyethylene glycol linker lengths to degrade a HaloTag-fused protein: very short linkers failed to induce degradation, whereas an optimal linker length (∼3 PEG units) enabled efficient ubiquitination and >95% depletion of the target (Zhang et al., 2025; Yossra Gharbi and Rocío Mercado, 2024). Such findings emphasize that the ternary complex geometry must be favorable – flexible linkers can allow the PROTAC to adopt a conformation that brings the target and E3 into a productive orientation. X-ray structures and computational modeling of ternary complexes have guided rational modifications to improve potency and selectivity. Moreover, PROTACs often suffer from large molecular weight and polar surface area, which can hinder cell permeability and oral bioavailability. Medicinal chemists have tackled this via prodrug strategies (e.g. mask charged groups with cleavable motifs) and by trimming unnecessary portions of linkers or capping polar handles. Despite these challenges, multiple PROTACs have achieved low-nanomolar or even picomolar cellular potency (Hartmann, 2025; Wang et al., 2024a), attesting to the power of an event-driven pharmacology (inducing a transient event leading to persistent protein knockdown). One nuance uncovered in recent years is that PROTACs can sometimes act as molecular glues in addition to their intended bifunctional mechanism. For instance, PROTACs employing pomalidomide (a CRBN ligand) were found to induce degradation of GSPT1, a neo-substrate of CRBN, even if GSPT1 was not the intended target – essentially behaving like a CRBN-based glue degrader (Verma et al., 2024; Lawer et al., 2024). Off-target degradation of IKZF1/3 transcription factors by some IMiD-based PROTACs has also been observed (Yu et al., 2025). This “glue-like” behavior arises when the E3 ligand portion of a PROTAC alone can induce degradation of native ligase substrates. Medicinal chemistry efforts are being made to mitigate such effects, for example by modifying the phthalate moiety of CRBN ligands to reduce recruitment of unintended substrates (Tomlinson et al., 2025a). Overall, PROTAC technology has matured significantly by 2025, with an expanding toolkit of E3 ligase ligands and linkers enabling tailored degraders. PROTACs represent a paradigm shift in organic drug design: rather than simply blocking protein function, chemists can create molecules that eradicate the protein, offering a powerful therapeutic approach especially for cancer, immunology, and other fields.

### 2.2 Molecular Glue Degraders

Molecular glue degraders are a distinct class of induced-proximity therapeutics that promote protein–protein interactions using a *single* small molecule rather than a bifunctional scaffold. Typically, a molecular glue binds to an E3 ubiquitin ligase (or, in some cases, the target protein) and creates a new composite interface that tethers the target to the ligase. This induced interaction leads to ubiquitination of the target and its subsequent proteasomal degradation. Many molecular glues were discovered serendipitously. A classic example is the plant hormone auxin, which was the first molecular glue described: auxin bridges the TIR1 ubiquitin ligase and Aux/IAA repressor proteins, thereby triggering their degradation (Tan et al., 2024). In humans, the immunomodulatory drugs (IMiDs) thalidomide, lenalidomide, and pomalidomide were later recognized as molecular glues. These compounds bind cereblon (CRBN) and remodel its substrate preference, recruiting specific zinc-finger transcription factors such as IKZF1 and IKZF3 for ubiquitination (Kumar and Nabet, 2024; Robinson and Banik, 2024). Thalidomide's teratogenicity and lenalidomide's efficacy in multiple myeloma are both explained by this neomorphic degradation of IKZF proteins (Kumar and Nabet, 2024). Another example is the aryl sulfonamide indisulam, which glues the RBM39 splicing factor to the DCAF15 substrate adaptor, leading to selective RBM39 degradation (Gregory et al., 2024). Together, these cases—once viewed as rare “black swan” events—demonstrated that small molecules can co-opt E3 ligases to degrade proteins that were previously considered “undruggable”. The identification and refinement of molecular glues are tightly intertwined with classical organic chemistry concepts such as structure–activity relationships (SAR), scaffold modification, and stereoelectronic control. The evolution from thalidomide to lenalidomide to pomalidomide illustrates how systematic structural variation and late-stage diversification of a common scaffold can dramatically alter E3 substrate specificity. Contemporary discovery approaches use parallel synthesis and fragment recombination to build focused libraries of analogues, which are then screened for glue-like activity. The pronounced sensitivity of molecular glues to small chemical changes underscores the importance of stereochemistry, heteroatom placement, ring substitution patterns, and physicochemical properties (e.g., polarity and hydrogen-bonding capacity). These parameters, which lie at the heart of organic and medicinal chemistry, ultimately govern whether a given scaffold can stabilize a productive ternary complex and function as an effective molecular glue degrader.

Lower path: Induced-proximity molecules co-opt this system. A PROTAC is a heterobifunctional molecule (two binding “warheads”) that simultaneously binds an E3 ligase and a target protein of interest (POI, blue), forcing formation of a ternary complex. This “hijacked” E3 ligase ubiquitinates the POI, leading to its proteasomal degradation (Alabi and Crews, 2021). A molecular glue degrader is a single small molecule that binds an E3 (often at an interface) and induces recruitment of a neo-substrate POI without needing two distinct warheads (Alabi and Crews, 2021). Both PROTACs and glues act catalytically, triggering ubiquitin-dependent degradation of the target; however, glues typically obey Lipinski's rules (<500 Da) and often emerge from phenotypic screens rather than rational design (Baisden et al., 2021; Banik et al., 2020). Notable examples include CRBN-binding immunomodulatory drugs like lenalidomide, which “glue” IKZF1/3 transcription factors to CRBN for ubiquitination (Ahn et al., 2021; Arimoto et al., 2021). This figure illustrates how both PROTACs and molecular glues leverage the cell's UPS to eliminate target proteins.

Recent years have seen a surge in identifying new molecular glue degraders and understanding their mechanism. A notable example is a series of compounds that bind to the kinase domain of CDK12/13 and simultaneously to the DDB1 adaptor of CRL4 ligase, thereby selectively degrading Cyclin K (the binding partner of CDK12/13) (Vetma et al., 2024a). These CDK12 inhibitors (e.g. CR8) unexpectedly function as glues – they do not contain two separate warheads, yet they induce a novel CDK12–DDB1 interaction leading to Cyclin K ubiquitination (Khaskia and Benhamou, 2025). Structural studies confirmed a ternary complex where the small molecule fills the ATP pocket of CDK12 and creates a surface that engages DDB1 (Haj-Yahia et al., 2023b). This discovery underscored that even weak or transient interactions can be exploited by a small molecule to “snap” two proteins together; many protein surfaces may be poised near a tipping point where a minor chemical perturbation induces binding (Verma, 2021). Encouraged by such findings, researchers are actively screening for glue degraders. Because glues often have subtle structure–activity relationships, traditional ligand-based design is challenging. Instead, phenotypic and genomic screening approaches have been fruitful. For instance, one strategy used genetically sensitized cells: by creating a cell line with impaired neddylation (mutation in UBE2M, which broadly inactivates Cullin–RING ligases), Winter and colleagues selectively identified compounds that lost activity in the mutant cells but were active in wild-type – these were enriched for candidate glue degraders (Hartmann, 2025; Wang et al., 2024b). Using this approach, they discovered a novel DCAF15-dependent glue that induces RBM23/RBM39 degradation (Xiao et al., 2025). Another innovative screen involved a cell painting assay to capture morphological changes: a custom library of CRBN-binding molecules was synthesized and tested for inducing characteristic phenotypes in cells overexpressing or lacking CRBN (Sarott et al., 2024). This led to identification of a CRBN glue that selectively degrades GSPT2 (a translation termination factor) (Dudas and Miteva, 2024). These examples highlight how chemical biology and functional genomics are guiding the discovery of glues beyond the known IMiDs and sulfonamides. From an organic chemistry standpoint, molecular glues often originate from subtle modifications of known bioactive scaffolds. For example, lenalidomide differs from thalidomide by a single substituent yet has enhanced efficacy and altered substrate spectrum. Minor changes in a ligand can “reprogram” a ligase's recruitment profile. This sensitivity poses a challenge in rationally designing glues, but it also offers opportunity: by iteratively tweaking a ligand's structure and profiling its effects (via proteomics or phenotypic assays), chemists can discover analogues with new degradation targets. There is also interest in rational glue design – for instance, using ternary complex structure modeling to design small molecules that could simultaneously contact two proteins. While this is still nascent, the success of recruiting multiple neo-substrates to CRBN and DCAF15 suggests a wide chemical space to explore (Gregory et al., 2024). Already, some companies have disclosed non-bifunctional degraders for targets like EGFR and mutant KRAS, presumably functioning as glues, although details remain proprietary. Molecular glues are attractive because they tend to be smaller and more drug-like than PROTACs (often <500 Da, obeying more of Lipinski's rules), and they can reach protein surfaces not addressable by conventional inhibitors. They blur the line between “ligand” and “catalyst”: a well-designed glue essentially turns the target into its own undoing by recruiting its executioner. As of 2025, molecular glue degraders have become an exciting complement to PROTACs together constituting a growing “toolkit” of proximity-inducing chemistries to control protein fate.

### 2.3 LYTACs: lysosome-targeting chimeras for extracellular protein degradation

PROTACs and most early TPD technologies are limited to intracellular proteins, since they rely on the ubiquitin–proteasome system. In contrast, lysosome-targeting chimeras (LYTACs) extend induced-proximity degradation to secreted and cell-surface proteins a huge portion of the proteome (hormones, cytokines, membrane receptors) that was previously out of reach (Tomlinson et al., 2025b; Song et al., 2024). LYTACs achieve this by a clever chemical strategy: they are typically bifunctional molecules (or conjugates) that bind a target protein on one end and, on the other end, bind to a cell-surface receptor that normally traffics to lysosomes. The most common receptors used are the cation-independent mannose-6-phosphate receptor (CI-M6PR) and the asialoglycoprotein receptor (ASGPR). CI-M6PR naturally binds mannose-6-phosphate (M6P) tags on enzymes for lysosomal delivery, while ASGPR binds glycoproteins with GalNAc sugars (primarily in hepatocytes). A LYTAC bearing an M6P glycan or GalNAc cluster will hitchhike on these receptors: it forms a ternary complex between the target and the receptor at the cell surface, the complex is endocytosed, and the target is delivered to lysosomes for degradation (the receptor itself can recycle) (Huang, 2024; Hinterndorfer et al., 2025). This concept was first demonstrated in 2020 by Bertozzi and colleagues, who synthesized conjugates of antibodies or peptides with multiple M6P residues (“M6Pn”) (Dong et al., 2021; Vetma et al., 2024b). For example, an anti-EGFR antibody was decorated with M6P glycopeptides, yielding a LYTAC that bound EGFR on cells and also bound CI-M6PR, dragging EGFR into lysosomes (Khaskia and Benhamou, 2025). They showed degradation of ∼30–70% of EGFR and HER2 on cancer cells depending on the antibody used (Yoon et al., 2024). Importantly, a LYTAC targeting apolipoprotein E4 (ApoE4, an extracellular protein implicated in Alzheimer's disease) successfully reduced ApoE4 levels, illustrating therapeutic potential beyond oncology (London, 2024a). Knockdown of CI-M6PR via CRISPR abrogated LYTAC activity, confirming that the mechanism requires receptor-mediated internalization (London, 2024a).

Since 2020, multiple flavors of LYTACs have been developed. One major advance was the creation of small-molecule LYTACs or MoDE-As (molecular degraders of extracellular proteins, class A) that use a synthetic tri-GalNAc cluster to engage ASGPR, instead of the bulky M6P-glycoproteins (Hartmann, 2025; Wang et al., 2024c). Because ASGPR is predominantly expressed in the liver, GalNAc-LYTACs can achieve tissue-specific degradation confined to hepatocytes (Hartmann, 2025). In 2021, researchers attached a tri-GalNAc tag to a synthetic peptide that binds integrins, producing a LYTAC that led to robust integrin degradation and consequent inhibition of cell proliferation (Xiao et al., 2025; Bonet‐Aleta et al., 2024). Similarly, a GalNAc-functionalized version of cetuximab (anti-EGFR) was as effective as the M6P-functionalized version in degrading EGFR, but required tenfold lower concentration in liver-derived cells due to the high ASGPR uptake efficiency (Xiao et al., 2025). Another variation used DNA aptamers in place of antibodies: aptamers against cancer cell surface proteins were conjugated to M6P or GalNAc moieties, yielding entirely nucleic-acid LYTACs that achieved target internalization and lysosomal degradation (Dudas and Miteva, 2024; Tan et al., 2024). Aptamer-based LYTACs are smaller and potentially less immunogenic than antibodies. A recent innovation is KineTACs, which utilize a peptide ligand (like CXCL12) to engage a decoy receptor (CXCR7) that is scavenged to lysosomes; a KineTAC targeting TNF-α via CXCR7 showed degradation of extracellular TNF-α, highlighting that even secreted ligands can be neutralized by induced proximity to lysosomes (Zhang et al., 2024). These developments underscore the creativity in LYTAC design – any ligand that can recruit a lysosomal routing receptor could, in principle, serve as the “delivery address” on a LYTAC molecule. deeply linked to synthetic conjugation chemistry. For LYTACs, multi-step synthesis of mannose-6-phosphate (M6P) glycopolymers, trivalent GalNAc motifs, and their attachment to antibodies or aptamers requires mastery of glycosylation reactions, solid-phase synthesis, and biorthogonal conjugation. AUTACs, by contrast, rely on the synthetic incorporation of guanine analog tags through stable linkers, using strategies such as reductive amination, carbamate linkages, and aromatic substitution. These synthetic methodologies not only provide molecular diversity but also enable fine-tuning of stability, solubility, and cell permeability, reinforcing the central role of organic chemistry in rendering these modalities drug-like.

LYTAC technology is noteworthy for its reliance on synthetic conjugation chemistry at the interface of small molecules and biologics. The original LYTACs required multi-step synthesis of M6P glycopolymers and site-specific bioconjugation to antibodies (Ng and Banik, 2022; Paiva and Crews, 2019). Subsequent approaches have explored more drug-like scaffolds – for instance, a synthetic polymer decorated with multiple M6P analogues was coupled to a small targeting ligand (like biotin or a peptide) via click chemistry (Paiva and Crews, 2019; Kim et al., 2022). Tri-GalNAc motifs have been incorporated through solid-phase synthesis or as pre-assembled modules attached via stable linkers. These advances make LYTACs more amenable to medicinal chemistry optimization (e.g., tuning spacer lengths, valency of glycan, stability in circulation). A pharmacokinetic consideration is that large bioconjugate LYTACs (antibody-based) may have limited tissue penetration, whereas smaller peptide or aptamer LYTACs could diffuse more readily but might be cleared faster by the kidneys. In all cases, because LYTACs target proteins to lysosomes (which degrade material more slowly than proteasomes), the kinetics of target removal may differ from PROTACs. Still, LYTACs fill a critical gap: they offer a way to drug the “undruggable” extracellular proteome. Already there is interest in using LYTACs to degrade pathogenic secreted proteins (like ApoE4, amyloid precursors) in neurodegenerative diseases, or to remove immune checkpoint proteins (like PD-L1) from the tumor cell surface, as an immunotherapy strategy (Ding et al., 2022; Ng and Banik, 2022; Paiva and Crews, 2019). As of 2025, LYTACs remain in preclinical stages, but their successful demonstrations have paved the way for translational efforts. Ongoing research aims to identify new lysosomal-targeting ligands (beyond CI-M6PR and ASGPR) to broaden the approach – for example, leveraging the sortilin receptor for neuronal uptake or others expressed in different tissues (Kim et al., 2022). The organic chemistry innovation in LYTACs namely, crafting bifunctionals that marry targeting ligands with glycoconjugates – exemplifies how chemists can repurpose the cell's endosome–lysosome system for therapeutic benefit.

### 2.4 AUTACs and autophagy-mediated degraders

Cells possess another major degradation route besides proteasomes and endocytosis: the autophagy–lysosome pathway, which can eliminate cytosolic proteins, aggregates, large macromolecular assemblies, and even entire organelles. In macroautophagy, selected cargo is sequestered into double-membraned vesicles (autophagosomes) that subsequently fuse with lysosomes, where the encapsulated material is degraded. Two innovative induced-proximity strategies have been developed to co-opt this pathway for targeted degradation: AUTACs (autophagy-targeting chimeras) and autophagosome-tethering compounds such as ATTECs (autophagy-tethering compounds) and AUTOTACs. In AUTACs, a degradative tag—often a guanylation-based or otherwise modified small-molecule motif—is covalently linked to a ligand for the protein of interest, thereby mimicking endogenous autophagy signals and promoting selective engulfment by autophagosomes. In contrast, ATTECs and AUTOTACs use bifunctional small molecules that bind both an autophagy-related component (e.g., LC3 or p62/SQSTM1) and the target, physically tethering the cargo to the autophagic machinery. Although they differ in the precise chemical tags and binding motifs employed, all of these strategies rely on rational organic design of linkers and recognition elements to convert the general autophagy–lysosome pathway into a programmable, small-molecule-controlled degradation system.

While the core concepts of induced-proximity modalities are now widely reviewed, their continued development critically depends on chemical innovation. Linker engineering has evolved from simple PEG spacers to more sophisticated designs, including cleavable motifs (esters, carbonates, disulfides), rigid scaffolds (triazoles, aryl spacers), and prodrug-inspired linkers that improve stability and pharmacokinetics. Ligand discovery, which was once largely serendipitous, now increasingly relies on DNA-encoded libraries, covalent ligand screening, and AI-guided molecular design, providing chemists with precise control over degrader scaffolds. From a medicinal chemistry standpoint, optimization of physicochemical properties through macrocyclisation, stereoelectronic tuning, and strategic polarity masking is emerging as a decisive factor for clinical translation. Across emerging technologies such as TransTACs, DUBTACs, and Nano-AUTACs, synthetic feasibility and linker modularity consistently appear as unifying themes, underscoring organic chemistry as a primary driver of innovation in this field.

**Fig. 3:**
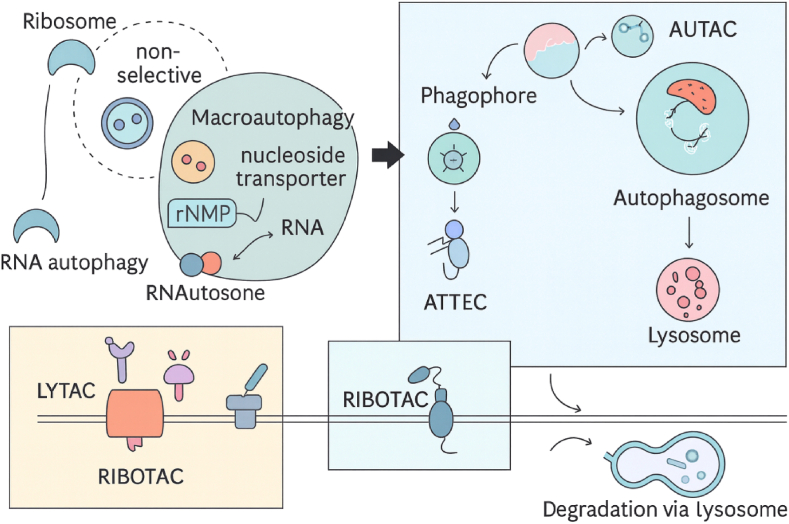
Engaging the endosomal-lysosomal pathway using LYTACs and MoDE-As. Both LYTACs and MoDE-As connect extracellular or membrane-associated proteins of interest (POIs) to a recycling receptor, promoting their internalization and subsequent degradation in the lysosome. LYTACs leverage the cation-independent mannose-6-phosphate receptor (CI-M6PR), a recycling membrane protein that interacts with POIs tagged with an MP6n label. In contrast, GalNAc-LYTACs and MoDE-As employ a GalNAc tag to target the asialoglycoprotein receptor (ASGPR), which is specific to the liver and facilitates lysosomal degradation in a tissue-selective manner (Ding et al., 2022). (Modified and redrawn by the authors).

**Fig. 4:**
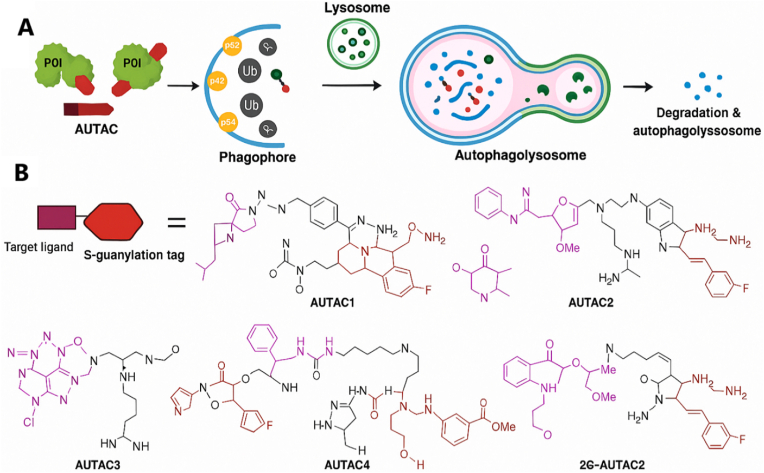
AUTACs: autophagy-targeting chimeras induce ubiquitin-dependent autophagic degradation (modified and redrawn based on Takahashi et al. (Takahashi et al., 2019)). (Modified and redrawn by the authors).

AUTACs were introduced in 2019–2020 by Arimoto and co-workers as small molecules that induce “self-tagging” of the target protein with a degradation signal (Banani et al., 2016; Bergeron-Sandoval et al., 2016). An AUTAC typically consists of a target-binding ligand covalently linked to an S-guanylation “degradation tag”, derived from guanine (p-fluorobenzylguanine, FBnG), which mimics a naturally occurring post-translational modification. This tag promotes installation of K63-linked polyubiquitin chains on the target, which are recognized by autophagy receptors such as p62/SQSTM1 (Bergeron-Sandoval et al., 2016). The ubiquitin-tagged protein is then captured by p62, which binds both ubiquitin and LC3 on the autophagosome membrane, thereby routing the cargo into autophagosomes and ultimately the lysosome for selective autophagy (Fig. 3). The chemical design of AUTACs was inspired by the observation that bacteria tagged with 8-nitro-cGMP, a small molecule structurally related to guanine, become selectively ubiquitinated and cleared by xenophagy (Feric et al., 2016). Arimoto's group synthesized a cell-permeable FBnG analog and showed that covalent attachment of FBnG to a protein is sufficient to trigger its autophagic degradation (Banani et al., 2016; Hyman et al., 2014). They then created AUTAC1 by linking FBnG to fumagillin, a covalent inhibitor of MetAP2, yielding a chimera that induced ∼80% degradation of MetAP2 in cells at micromolar concentrations (Feric et al., 2016; Hyman et al., 2014). A non-covalent AUTAC2 targeting FKBP12 also efficiently degraded its target, demonstrating that the concept is compatible with both covalent and non-covalent warheads (Cavey et al., 2005). AUTACs have even been applied to organelle-level degradation: by expressing a HaloTag on the mitochondrial outer membrane and treating with a HaloTag–ligand–FBnG hybrid, the same group induced mitophagy and clearance of dysfunctional mitochondria (Zaffagnini et al., 2018). Thus, AUTACs leverage synthetic “degradation tags” to redirect the ubiquitin system toward autophagy; although the precise molecular mechanism by which FBnG promotes K63 ubiquitination is still under investigation (Pikkarainen et al., 2011; Bayraktar et al., 2016), its functional efficacy is well established. ATTECs and AUTOTACs represent complementary autophagy-based strategies in which bifunctional small molecules directly tether the target to an autophagy organelle or receptor. ATTECs (autophagosome-tethering compounds) are heterobifunctional molecules that bind the target on one end and LC3, a core autophagosome protein, on the other (Marshall et al., 2016). In doing so, an ATTEC effectively “piggybacks” the target onto the growing autophagosome, promoting its engulfment and lysosomal degradation (Fig. 4A). The first ATTECs were reported in 2019 by Lu and colleagues, who identified compounds that bind both LC3 and mutant huntingtin (mHTT) with an expanded polyglutamine tract. These ATTECs selectively removed mHTT aggregates while sparing wild-type HTT (Cohen-Kaplan et al., 2017; Lim et al., 2015), and in Huntington's disease mouse models they reduced mHTT levels and improved phenotypes without obvious toxicity (Li et al., 2019b). Notably, these ATTECs were relatively small (<500 Da) and did not possess a classical “linker” in the PROTAC sense; instead, they embodied pharmacophores capable of engaging both proteins simultaneously (Fu et al., 2021). Subsequent work extended ATTECs to other cargos, such as lipid droplets, by combining an LC3-binding module with a lipid droplet–binding dye to drive lipophagic clearance of excess lipid in obese mice (Fu et al., 2021; Qian et al., 2025). These LD-ATTECs reduced pathological lipid accumulation in the liver and retina and ameliorated disease phenotypes in macular degeneration models (Qian et al., 2025; Zhang et al., 2023), highlighting a unique advantage of autophagy-mediated degraders: the ability to dispose of large, aggregated, or non-protein substrates that are inaccessible to the proteasome (Gnanaguru et al., 2021; Zhong et al., 2024). AUTOTACs, introduced around 2022, take a different approach by recruiting the endogenous autophagy receptor p62 (SQSTM1). P62 contains a ZZ domain that recognizes specific small-molecule motifs. AUTOTACs are designed as bifunctional molecules that couple a p62-binding ligand to a target-binding ligand (Vainshtein and Grumati, 2020). By engaging both p62 and the target, an AUTOTAC promotes p62 oligomerization and delivery of the cargo to autophagosomes via LC3 interaction (Vainshtein and Grumati, 2020). Ji et al. demonstrated AUTOTACs capable of degrading diverse proteins, including ERα, AR, and Tau, thereby establishing this as a general strategy parallel to ATTECs (Alam et al., 2025). Because p62 can also bind ubiquitin, some AUTOTAC designs may trigger ubiquitination as part of the degradation process, leading to mechanistic crosstalk between autophagy and ubiquitin-mediated signaling. The broader term “MADTAC” (macroautophagy degradation-targeting chimera) is sometimes used as an umbrella label for AUTAC, ATTEC, AUTOTAC, and related autophagy-directing chemistries (Ji et al., 2022; Zhao et al., 2022).

(A) Schematic mechanism of AUTAC action. A bifunctional AUTAC molecule consists of a target-binding ligand (purple) linked to an S-guanylation tag (red) that mimics 8-nitro-cGMP. Binding of the AUTAC to the protein of interest (POI, green) causes the POI to be modified with K63-linked ubiquitin chains (yellow “Ub”), attracting the autophagy receptor p62. P62 binds both ubiquitin and LC3 on the nascent autophagosome (phagophore), thereby recruiting the tagged POI into the autophagosome for degradation. The autophagosome eventually fuses with a lysosome (forming an autophagolysosome) where the POI is broken down. (B) Chemical structures of representative AUTAC molecules (AUTAC1–4 and 2G-AUTAC2) reported by Arimoto's group (Takahashi et al., 2019). Each contains a guanine derivative tag (red) and various target ligands (purple/black) such as a MetAP2 inhibitor (AUTAC1) or an FKBP ligand (AUTAC2). These structures highlight the medicinal chemistry involved in creating bifunctionals that are cell-permeable and retain binding on both ends while carrying the bulky guanine tag.

Autophagy-based degraders broaden the scope of induced-proximity chemistry, but they also come with distinct considerations. Kinetically, autophagy is a slower process than proteasomal degradation; thus, AUTACs/ATTECs might not achieve rapid protein knockdown but can handle persistent aggregates and larger targets. Selectivity is a key issue – for example, AUTACs rely on the cell's ubiquitination machinery which could, in theory, ubiquitinate off-target proteins if the tag is too promiscuous. So far, the designed tags (like FBnG) appear to be selective in context, but the detailed mechanisms need further elucidation (Komachankandy et al., 2025). Medicinal chemistry optimizations are ongoing to improve the potency of these molecules (many first-generation AUTACs/ATTECs required micromolar concentrations). Another challenge is monitoring their activity: traditional readouts like proteasomal inhibition or ubiquitin accumulation may not apply, so researchers use microscopy and degradation of fluorescent-tagged substrates to confirm autophagy involvement. Importantly, autophagy pathways can be induced by cellular stress, so any small molecule introduced must be vetted for not simply causing bulk autophagy upregulation (which could confound target-specific effects). The field has addressed this by showing that, for instance, an ATTEC does not degrade unrelated proteins or activate global autophagy markers unless the target is present (Dey and Jaffrey, 2019). In terms of therapeutic applications, autophagy tethering is particularly attractive for neurodegenerative diseases characterized by protein aggregates (tau, synuclein, huntingtin) and for conditions with toxic accumulations (e.g. fatty liver, as demonstrated with LD-ATTECs (Qian et al., 2025)). Overall, the period 2020–2025 has established autophagy-based induced proximity as a viable and versatile strategy. From an organic chemistry viewpoint, these molecules illustrate ingenious design – incorporating endogenous signaling cues (like guanine tags or LC3 ligands) into chimeric structures to redirect cellular disposal systems. As our understanding of autophagy's signals improves, we can expect even more refined AUTACs and related degraders to enter the scene.

### 2.5 RIBOTACs: ribonuclease-targeting chimeras for RNA degradation

Expanding the induced-proximity paradigm beyond proteins, researchers have recently developed small-molecule chimeras that target RNA for degradation. These are called RIBOTACs (ribonuclease-targeting chimeras) – heterobifunctional or multispecific molecules that bind an RNA of interest and simultaneously recruit an RNase to cleave that RNA (Costales et al., 2020). The motivation is clear: many disease drivers are RNAs (oncogenic mRNAs, viral RNAs, pathogenic non-coding RNAs) which might be hard to block with traditional approaches, but destroying them outright could be highly therapeutic. Nature already uses this strategy via antisense oligonucleotides and siRNAs that recruit RNase H or RISC to cut target RNA, but those modalities involve large nucleic acids. RIBOTACs offer a fully small-molecule approach to induce RNA degradation.

Most RIBOTACs to date harness the cell's RNase L as the executing nuclease. RNase L is part of the innate immune response, typically activated by binding of 2′,5′-linked oligoadenylates (2–5A) to its allosteric sites, leading to dimerization and cleavage of viral and cellular RNAs. In 2019, Disney and coworkers reported the first RIBOTACs: they attached a small molecule that binds a disease-causing RNA to a ligand known to bind and activate RNase L (Dey and Jaffrey, 2019; Zhang et al., 2021). One early example targeted pre-miR-21 (precursor of the oncogenic microRNA-21). A small molecule that selectively binds a structural bulge in pre-miR-21 was conjugated to an RNase L-recruiting module. The resulting RIBOTAC induced the selective cleavage of pre-miR-21, lowering miR-21 levels and phenocopying an antisense oligo against miR-21 (Haj-Yahia et al., 2023a). Importantly, in cells treated with the RIBOTAC, knockdown of RNase L (via siRNA or CRISPR) rescued the RNA levels, proving that RNase L was the mediator of degradation (Sui et al., 2019; Zheng et al., 2012). This mechanism was shown to be catalytic: a RIBOTAC can lead RNase L to cut the target at one site, then presumably RNase L dissociates and can engage another target molecule. Disney's group extended this concept to other targets: for instance, a RIBOTAC against the microRNA-155 precursor (pre-miR-155) was developed and shown to reduce mature miR-155 by >70% in breast cancer cells, with accompanying effects on miR-155-driven gene networks (Zheng et al., 2012). Likewise, they created RIBOTACs targeting an IRES element in the MYC oncogene mRNA, leading to site-specific cleavage of MYC RNA and suppression of Myc protein production (Rai et al., 2008; Thoma et al., 2008). These RIBOTACs have demonstrated on-target cellular activity without triggering a global interferon response (since RNase L is locally activated at the RNA of interest rather than systemically).

From an organic chemistry perspective, RIBOTACs exemplify the modular design of bifunctional molecules where RNA-binding scaffolds are coupled with RNase L recruiters. This requires synthetic flexibility, as many RNA binders originate from heteroaromatic scaffolds optimized via cross-coupling reactions (Suzuki, Buchwald–Hartwig) and late-stage functionalization. The RNase L-recruiting moieties, identified via DNA-encoded library screening, are incorporated through cleavable linkers or stable amide bonds. Iterative SAR efforts such as altering heterocyclic substitution or linker polarity determine whether the molecule achieves selective RNA engagement versus off-target binding. The ability to synthetically merge two distinct pharmacophores into a single chimeric entity reflects the ingenuity of modern organic synthesis.

**Fig. 5:**
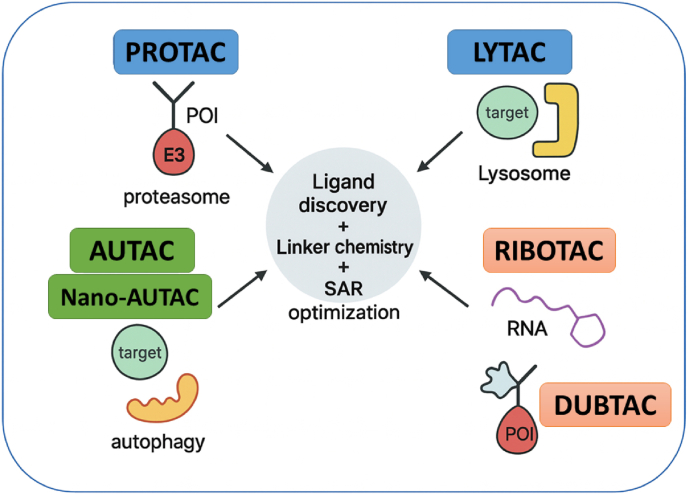
Comparative overview of induced-proximity modalities and their degradation pathways. This schematic illustrates the distinct mechanisms employed by various therapeutic agents: PROTACs facilitate targeted degradation via the proteasome, LYTACs promote lysosomal degradation, AUTACs engage autophagy processes, and RIBOTACs enable selective RNA degradation. Additionally, a timeline highlights the evolution of PROTAC technology, showcasing the transition from peptide-based constructs to small molecule PROTACs, the incorporation of click-linker strategies, and the discovery of novel covalent E3 ligands, emphasizing the organic chemistry perspective in the development of these innovative therapeutics. (Figure prepared by the authors using BioRender.

PROTAC (Proteolysis Targeting Chimeras): This bifunctional molecule consists of a ligand that binds to an E3 ligase and another ligand that targets a specific POI. By bridging these two components, PROTACs facilitate the ubiquitination and subsequent degradation of the target protein via the proteasome. LYTAC (Lysosome-Targeting Chimeras): LYTACs are designed to link extracellular or membrane-bound targets to lysosome-trafficking receptors. This modality enables the degradation of proteins that are not easily accessible to intracellular degraders, promoting targeted lysosomal degradation. RIBOTAC (RNA-Binding Oligonucleotide Targeting Chimeras): RIBOTACs utilize a small molecule that binds to RNA and recruits RNase for selective RNA degradation. This approach opens new therapeutic avenues by targeting RNA transcripts, expanding the scope of degradation strategies beyond proteins. AUTAC (Autophagy-Targeting Chimeras): AUTACs engage the autophagy machinery by linking target proteins to autophagy receptors through specific tags. This modality is particularly useful for clearing cellular aggregates and organelles, making it relevant in neurodegenerative diseases. DUBTAC (Deubiquitinase-Targeting Chimeras): DUBTACs are designed to recruit deubiquitinases to stabilize proteins, effectively reversing the ubiquitination process. This modality can restore the function of proteins that have been misregulated due to ubiquitin-mediated degradation. Fig. 5 also emphasizes the importance of ligand discovery, linker chemistry, and structure-activity relationship (SAR) optimization in the design and development of these innovative therapeutic agents. By illustrating the intricate relationships between these modalities and their respective mechanisms, the diagram underscores the potential of induced-proximity therapeutics in advancing targeted degradation strategies in drug discovery and development.A key challenge in designing RIBOTACs is finding a small molecule that can bind the RNA target with high affinity and selectivity RNA is a floppy, polyanionic polymer, and many small molecules that bind RNA do so nonspecifically. To address this, researchers have leveraged RNA-focused libraries and structure-based design. Disney's team employed a high-throughput screening of a “library-against-library”: thousands of small molecules were screened against a library of diverse RNA secondary structure motifs to identify novel RNA-binding chemotypes (Tong et al., 2023) (Tran and Disney, 2012). This yielded multiple scaffolds with specific RNA motif preferences (e.g., benzene derivatives that bind bulges, or azole-containing compounds for internal loops) (Tran and Disney, 2012) (Velagapudi et al., 2012). These were then computationally matched to motifs present in target RNAs of interest (like microRNA precursors). By this route, they discovered small molecules that bound pre-miR-155 and the Myc IRES with nM affinity (Lam et al., 2025) (Zhang et al., 2021). On the RNase L side, medicinal chemists have sought small, non-oligonucleotide ligands that can recruit or activate RNase L. A breakthrough came when a DNA-encoded library (DEL) screen against RNase L identified a series of 2-aminothiazole and thienopyrimidinone compounds that bind to the RNase L pseudokinase domain (Takashima et al., 2022). Attaching these RNase L ligands to RNA-binding modules produced effective RIBOTACs (Huang et al., 2014) (Childs-Disney et al., 2022). For example, a thienopyrimidinone-based recruiter was conjugated to a pre-miR-155 binding molecule to create the aforementioned pre-miR-155-RIBOTAC (Zhang et al., 2021) (Sui et al., 2019). The modular design is reminiscent of PROTAC assembly, except one “warhead” recognizes an RNA structure instead of a protein surface. PROTACs are bifunctional compounds that utilize the body's natural protein degradation mechanisms, specifically the ubiquitin-proteasome system, to selectively target and eliminate proteins associated with diseases. Similar to their mythological three-headed counterpart, a PROTAC is composed of three key structural elements (see Fig. 5), a ligand that interacts with the ubiquitin-protein ligase E3 (referred to as the E3 ligase recognition component), a ligand that attaches to the protein intended for degradation, and a covalent linker that connects these two ligands. By facilitating the proximity of the E3 ligase and the target protein, PROTACs enable the E3 ligase to tag the target protein with ubiquitin, thereby promoting its degradation. Once the target protein is degraded, the PROTAC molecules are released.

One major concern with RIBOTACs is off-target cleavage of unintended RNAs. RNase L shows some sequence preference – it cleaves single-stranded regions, often after UU or UA dinucleotides – but broadly activated RNase L can degrade many transcripts, which underlies its antiviral effect. Therefore, an effective RIBOTAC must localise RNase L to the intended RNA target without triggering global RNase L activation. In practice, the recruited RNase L in these chimeras appears to act locally. For example, transcriptome-wide sequencing in cells treated with a pre-miR-155.

**Fig. 6:**
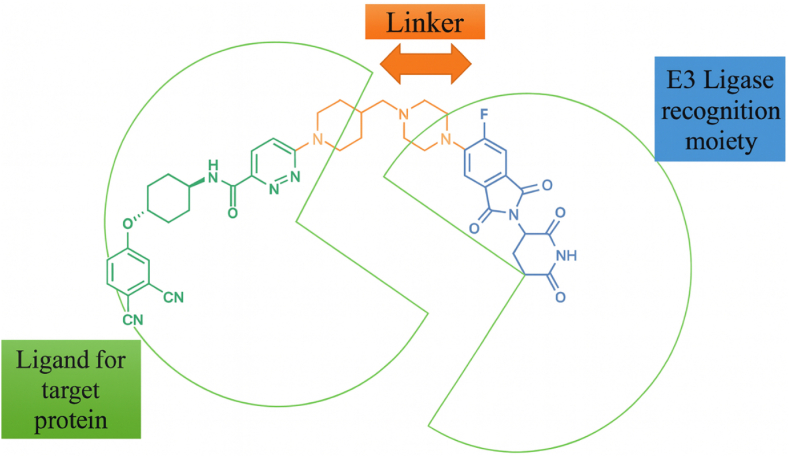
Schematic representation of a typical PROTAC structure, highlighting its bifunctional nature. The PROTAC consists of three main components: a ligand that binds to the E3 ligase, a ligand that targets the protein of interest (POI), and a linker that connects these two ligands. An example of a PROTAC, ARV-110, is shown, demonstrating its structural features and functional groups that facilitate the targeted degradation of specific proteins through the ubiquitin-proteasome system. (Figure prepared by the authors using BioRender/ChemDraw/powerpoint.

RIBOTAC showed a selective reduction of miR-155 with minimal off-target RNA changes (Loeb et al., 2012; Xu et al., 2010; Ursu et al., 2020). Knockdown of RNase L abolished these effects, confirming that the observed RNA degradation is both RNase L–dependent and target-specific (]). To further increase precision, several conditional RIBOTAC designs have been developed (Fig. 6). One approach is photoactivation: Hargrove, Disney and co-workers reported a “photoactivated” RIBOTAC in which the RNase L–recruiting module is caged by a photolabile group; upon light irradiation the cage is removed, the molecule becomes active, and local RNA cleavage is induced, allowing spatial and temporal control [ref]. Another strategy uses prodrug RIBOTACs that are unmasked by tumour-enriched enzymes, thereby restricting RNase L activation to diseased tissue. These refinements aim to minimise unwanted RNA degradation in healthy cells and concentrate the effect in pathological contexts.

While each modality (PROTACs, molecular glues, LYTACs, AUTACs, and RIBOTACs) has been described individually, their critical comparison highlights both shared principles and unique challenges. At a design level, PROTACs and molecular glues both exploit the ubiquitin–proteasome system but differ in synthetic complexity: PROTACs demand multistep bifunctional assembly and precise linker optimization, whereas glues emerge from subtle scaffold modifications and SAR exploration. LYTACs extend degradation to extracellular proteins but rely heavily on glycosylation and bioconjugation chemistry, which introduces challenges of synthetic accessibility and pharmacokinetics. AUTACs and ATTECs leverage autophagy, enabling clearance of aggregates and organelles, yet their chemical design is constrained by the need to incorporate bulky guanine tags or LC3-binding motifs without compromising cell permeability. RIBOTACs, in turn, face the dual hurdle of synthesizing selective RNA-binding scaffolds and chemically coupling them to nuclease recruiters, a task that demands sophisticated heteroaromatic chemistry and linker engineering. From a comparative standpoint, the relative strengths and weaknesses of these approaches can be summarized as follows:

PROTACs: High potency, catalytic degradation; challenged by large size and poor oral bioavailability (see Table 1). Molecular glues: Small, drug-like scaffolds; but limited rational design rules and potential off-target effects. LYTACs: Enable extracellular targeting; synthetic complexity and tissue penetration remain limiting. AUTACs/ATTECs: Address aggregates and organelles; slower kinetics and linker permeability are hurdles (see Table 2) (Mikutis and Bernardes, 2024). RIBOTACs: Open RNA as a therapeutic frontier; selectivity and RNA-binding chemistry remain underdeveloped. Together, these comparisons underscore that the unifying organic chemistry principle is modularity: synthetic design must balance feasibility, stability, and selectivity across diverse biological contexts. Recognizing these trade-offs provides a more integrated framework for future degrader development.

**Table 1:** Emerging induced-proximity technologies and their organic-chemistry features (Aguzzi and Altmeyer, 2016). [60]

Technology	Mechanism	Distinctive Features	Key Organic Chemistry Principles
TransTAC (Transmembrane-targeting chimera)	Binds extracellular target + recruits a transmembrane receptor for lysosomal routing	Targets proteins not accessible to intracellular degraders	Antibody–ligand conjugation; biorthogonal click chemistry; linker stability in membranes
GlueTAC	Combines glue-like recruitment with lysosomal degradation	Single small molecule induces ternary complex with lysosome-trafficking receptor	Scaffold modifications to tune glue activity; small-molecule glycosylation
DUBTAC (Deubiquitinase-targeting chimera)	Recruits a deubiquitinase to stabilize proteins	Opposite of PROTAC; restores loss-of-function proteins	Modular linker synthesis; attachment of covalent/non-covalent DUB ligands
Nano-AUTACs	Nanoparticle-decorated guanine tags promote autophagy	Improves pharmacokinetics and tumor selectivity	Supramolecular assembly; surface functionalization; multivalent conjugation

**Table 2:** Comparative analysis of induced-proximity modalities from an organic-chemistry design perspective.

Modality	Key design principles	Strengths	Weaknesses/limitations	Synthetic & chemical challenges
PROTACs	Modular warhead–linker–E3 ligase ligand architecture; control of linker length, orientation, and rigidity to favour productive ternary complex formation.	Catalytic degradation; broad target scope; existence of clinical-stage examples (see Table 3).	Large size, high polarity, and poor oral bioavailability; potential off-target ‘molecular glue’ effects.	Multistep synthesis; extensive linker optimization (length, polarity, rigidity); protecting-group strategies; introduction of chiral centres; polarity-masking or macrocyclisation strategies.
Molecular glues	Single small-molecule scaffold derived from an E3 ligase ligand; subtle modifications create a new protein–protein interface between ligase and POI.	Drug-like size and physicochemical properties; simple scaffold; catalytic mode of action.	Limited rational design rules; discovery often serendipitous; potential off-target substrate recruitment.	Fine, often non-intuitive SAR; scaffold hopping; late-stage functionalization to tune specificity and ligase/substrate selectivity.
LYTACs	Bifunctional conjugates linking target binders (antibodies or small molecules) to lysosome-trafficking ligands (e.g., M6P, GalNAc) via defined bioconjugation linkers.	Enable degradation of extracellular and membrane proteins; possibility of tissue selectivity through receptor choice.	Bulky constructs (often antibody-based); limited tissue penetration; potential immunogenicity and complex PK.	Multi-step glycan synthesis; solid-phase or solution-phase preparation of M6P/GalNAc motifs; site-specific and homogeneous bioconjugation to antibodies or small-molecule binders.
AUTACs/ATTECs/AUTOTACs	Small molecules or bifunctionals that attach degradation tags (e.g., guanine-based tags, LC3-binding motifs, hydrophobic tags) to POIs or organelles to recruit autophagy machinery.	Unique ability to clear aggregates, damaged organelles and lipids; attractive for neurodegenerative and metabolic diseases.	Slower kinetics than proteasomal degradation; limited efficacy on nuclear targets; requirement for cell-permeable bulky tags.	Incorporation of guanine analogues or LC3-binding pharmacophores; installation of bulky autophagy tags while maintaining permeability; optimization of linker stability and metabolic liability.
RIBOTACs	RNA-binding small-molecule scaffold tethered to an RNase-recruiting ligand (e.g., RNase L agonist) via a suitable linker.	Opens disease-relevant RNA as a degradable target class; potential for high sequence and structure selectivity.	Challenging selectivity; risk of unintended RNA cleavage and global translational effects; currently at preclinical stage.	Design of selective RNA-binding scaffolds (heteroaromatic frameworks, fragment-based ligands); modular coupling to RNase ligands via amide or cleavable linkers while retaining binding affinity and drug-like properties.

While still early-stage, RIBOTACs exemplify the power of organic chemistry to bridge two biomolecules that normally would not interact (a small molecule binding an RNA and an enzyme) to achieve a functional outcome. They effectively create an “RNA degrader” in analogy to protein degraders. The years 2020–2025 have seen RIBOTACs applied in cell models of cancer, infectious disease (targeting viral RNAs), and repeat expansion disorders (targeting toxic RNA repeats) (Haj-Yahia et al., 2023b). For example, a RIBOTAC was designed to target the RNA G4C2 repeats implicated in C9orf72 ALS/FTD, and it reduced the accumulation of toxic repeat RNA foci in patient-derived cells (this used RNase H recruitment via a DNA oligonucleotide-RNase H ligand conjugate, showcasing alternative nucleases for RNA degradation) (London, 2024b). As medicinal chemists continue to improve cell permeability and stability of RIBOTAC molecules, the prospect of drug-like small molecules that can eliminate pathological RNAs is coming into view. The specificity of RNA recognition is a challenge – nature solves it with antisense base-pairing, but small molecules require exploitable 3D motifs. Advances in RNA structural biology and computational docking are helping identify such pockets. It is notable that RIBOTACs build on decades of chemical biology targeting RNA (e.g., aminoglycosides binding rRNA, etc.), repurposing that knowledge for degradation instead of simply inhibition. In summary, RIBOTACs extend induced-proximity therapeutics into the realm of RNA, marking an exciting frontier where the lines between “chemistry” and “biology” blur: synthetic molecules are orchestrating the selective destruction of nucleic acids inside cells, much as siRNAs or CRISPR guides do, but through a wholly chemical agent.

This figure illustrates the mechanism by which LYTACs (Lysosome-Targeting Chimeras) and MoDE-As (Molecular Degraders of Extracellular Proteins) exploit the endosomal-lysosomal pathway to facilitate the degradation of extracellular or membrane-bound proteins of interest (POIs). Mechanism Overview: LYTACs and MoDE-As function by tethering POIs to specific recycling receptors on the cell membrane (see Table 3). This interaction promotes the internalization of the POIs, leading them to the lysosome where they undergo degradation. LYTACs: These molecules utilize the cation-independent mannose-6-phosphate receptor (CI-M6PR), a well-characterized recycling membrane protein. LYTACs are designed to bind to POIs that have been modified with an MP6n tag, allowing for efficient recognition and internalization. MoDE-As: In contrast, MoDE-As employ a GalNAc tag to recruit the asialoglycoprotein receptor (ASGPR). This receptor is specific to liver cells and plays a crucial role in the targeted degradation of proteins within this tissue. The use of the GalNAc tag ensures that the degradation process is tissue-specific, enhancing the therapeutic potential of MoDE-As. Implications: The ability of LYTACs and MoDE-As to direct proteins to the lysosome for degradation represents a significant advancement in targeted protein degradation strategies. This approach not only allows for the removal of unwanted proteins but also offers a novel method for therapeutic interventions in various diseases, particularly those involving extracellular proteins.

**Table 3:** Induced-proximity therapeutic modalities, their targets, recruited effectors, and degradation/modulation pathways.

Modality	Targeted biomolecule	Effector recruited	Degradation/modulation pathway	Example molecules
PROTACs	Intracellular proteins	E3 ubiquitin ligase	Ubiquitin–proteasome system	ARV-110, ARV-471
Molecular glues	Intracellular proteins	E3 ubiquitin ligase	Ubiquitin–proteasome system	Thalidomide analogues
LYTACs	Extracellular/secreted proteins	CI-M6PR, ASGPR	Lysosomal degradation	M6P–antibody conjugates
AUTACs	Aggregated/damaged proteins, organelles	Autophagy receptors (e.g., p62)	Autophagy–lysosome pathway	AUTAC4
ATTECs	Organelles (e.g., mitochondria, lipid droplets)	LC3	Autophagy–lysosome pathway	ATTEC1
RIBOTACs	RNA transcripts	RNase L	Targeted RNA degradation	RIBOTAC-FTX
DUBTACs	Intracellular proteins	Deubiquitinase	Protein stabilization	DUBTAC–OTUB1
TransTACs	Membrane proteins	Transmembrane receptors	Receptor internalization/lysosome trafficking	Antibody–ligand conjugates

## 3 Organic-chemistry challenges and design strategies across degrader modalities

Degrader modalities share the common conceptual feature of induced proximity, yet they pose distinct challenges from a synthetic and organic-chemistry perspective. PROTACs. For PROTACs, the key synthetic hurdles arise from their large size and highly polar, multifunctional architecture. Assembling two complex ligands and a linker often results in long, low-yielding linear routes, and the introduction of multiple stereocentres in the warhead and E3 ligase ligand further complicates scale-up. To address these issues, most programmes rely on convergent synthetic strategies in which the warhead and ligase ligand are prepared separately and coupled in the final steps via robust amide or click-type linkages. Recent work has also explored polarity-masking prodrugs and macrocyclisation strategies to improve cell permeability and oral exposure while maintaining degrader potency. Molecular glues. In contrast, molecular glues are usually single small molecules, but the synthetic challenge lies in extremely subtle and often non-intuitive structure–activity relationships. Small scaffold modifications can dramatically change which substrates are recruited, or abolish glue activity altogether. Discovery therefore frequently starts from serendipitous hits, followed by scaffold hopping and late-stage functionalization campaigns to tune ligase and substrate selectivity. From a chemistry standpoint, having modular points of diversification on the glue scaffold is crucial to enable rapid exploration of exit vectors, hydrogen-bond donors/acceptors, and aromatic substitution patterns.

LYTACs. LYTACs occupy an intermediate space between biologics and small molecules. They typically rely on glycan-based ligands (such as M6P or GalNAc clusters) conjugated to antibodies or small-molecule binders of the target. The main synthetic burdens are multi-step glycan synthesis, control of glycan valency and spacing, and achieving site-specific, homogeneous bioconjugation to the targeting moiety. Orthogonal protection strategies and bioorthogonal coupling reactions (e.g., azide–alkyne cycloadditions, oxime formation) are commonly used to introduce lysosome-trafficking motifs at defined sites without compromising target binding.

AUTACs, ATTECs and related autophagy-directed degraders. AUTACs and ATTECs use small-molecule tags to engage autophagy receptors or LC3, enabling clearance of aggregates, damaged organelles and lipids. The synthetic challenge is to introduce relatively bulky autophagy tags – such as guanine-based motifs, LC3-binding pharmacophores or hydrophobic tags – while preserving overall cell permeability and metabolic stability. Chemists therefore seek minimal tags that still engage the autophagy machinery, carefully choosing the attachment point on the warhead and optimizing linker length and polarity so that the resulting molecules remain within a tractable property space.

RIBOTACs. RIBOTACs extend induced-proximity concepts to RNA, combining an RNA-binding small-molecule scaffold with a ligand for RNase L or an alternative nuclease. From an organic-chemistry viewpoint, the most demanding step is the creation of selective RNA-binding scaffolds that can discriminate a single transcript against a backdrop of highly similar RNA structures. This typically requires heteroaromatic frameworks with precise three-dimensional shape and hydrogen-bonding patterns, often identified through fragment-based or diversity-oriented synthesis. The RNA binder must then be tethered to the nuclease-recruiting ligand via an amide or cleavable linker without disrupting either binding event, which imposes stringent constraints on linker length, orientation and attachment points.

DUBTACs, TransTACs and related emerging platforms. Newer induced-proximity concepts such as DUBTACs, TransTACs and nano-AUTACs bring additional synthetic complexity. DUBTACs require covalent or high-affinity non-covalent ligands for specific deubiquitinases, which are still relatively underexplored from a medicinal-chemistry standpoint, as well as modular linkers that do not interfere with enzyme activity. TransTACs and GlueTACs integrate antibody or protein components with small-molecule ligands, making site-specific conjugation and control of linker stability in the membrane environment critical. Nano-AUTACs add another layer of supramolecular design, in which surface functionalization of nanoparticles with multiple degrader tags must be carefully optimized to balance potency, pharmacokinetics and manufacturability.

## 4 Linker chemistry and structure–property relationships

Across degrader modalities, linker design plays a central role in translating binding affinity into efficient ternary complex formation and favorable pharmacokinetic properties. Different linker classes – rigid, flexible, cleavable, macrocyclic and polarity-masking – address distinct chemical and biophysical constraints.

Rigid linkers. Rigid linkers, such as aryl–aryl spacers, alkynes or other conformationally constrained motifs, reduce the entropic penalty associated with ternary complex formation by pre-organising the relative orientation of the binding elements. In PROTACs, replacing a fully flexible PEG chain with a semi-rigid aryl or alkynyl segment can dramatically increase degradation efficiency by favouring a productive geometry of the E3 ligase–PROTAC–POI complex. The disadvantage is that rigid linkers are often narrow in their SAR: small changes in length or angle can abolish degradation, necessitating careful, structure-guided optimization.

Flexible linkers. Flexible linkers (e.g., alkyl or PEG chains) provide conformational freedom, allowing the degrader to sample multiple binding geometries and tolerate movement between the ligase and target. This flexibility can be advantageous when structural information is limited. However, highly flexible and polar linkers may increase the entropic cost of ternary complex formation, broaden the spectrum of off-target complexes, and adversely impact pharmacokinetics by increasing molecular weight and topological polar surface area.

Cleavable linkers. Cleavable linkers introduce an additional control element, particularly useful when systemic exposure or tissue selectivity is a concern. Disulfide, ester, acetal, or enzyme-labile linkers can be designed to remain stable in plasma but to fragment in the intracellular or lysosomal environment. In PROTACs and LYTACs, such linkers can release a more compact active species once inside the cell or after internalization, thereby improving pharmacokinetics and potentially reducing long-lived circulating high-molecular-weight constructs.

Macrocyclic and constrained linkers. Macrocyclisation has emerged as a strategy to reconcile high affinity with improved permeability in large, polar molecules. Constraining part of the linker into a macrocycle can reduce conformational flexibility, shield polar groups, and pre-organise the degrader for ternary complex formation. For PROTACs, macrocyclic variants have shown that carefully chosen ring closures can maintain or enhance degradation potency while giving more drug-like absorption and clearance profiles.

Polarity-masking and prodrug linkers. Finally, polarity-masking linkers and prodrug concepts aim to temporarily hide hydrogen-bond donors and acceptors to facilitate membrane permeation. Examples include intramolecular hydrogen bonding, cyclic carbonates or carbamates, and enzymatically cleavable promoieties attached to polar linker segments. Once inside the cell, these masks are removed, regenerating the highly polar degrader capable of engaging both the ligase and the target. Such strategies are particularly relevant for PROTACs and autophagy-targeting degraders, where the inherent polarity of the scaffold is a major liability.

Together, these linker strategies illustrate how relatively small organic-chemistry changes in the spacer region can strongly influence ternary complex stability, degradation efficiency and in vivo pharmacokinetics, and therefore represent a key lever for future degrader optimization.

## 5 Conclusion and outlook

Unlike many existing reviews that catalog induced-proximity technologies, our perspective emphasizes the chemical logic that unites and differentiates them. The future of targeted degradation will be shaped not only by identifying new biological pathways, but also by chemists' ability to design modular scaffolds, introduce novel linker motifs, and reprogram effector selectivity through rational chemical design. The comparative analysis provided here underscores that each modality carries unique synthetic challenges—from glycosylation chemistry in LYTACs to heteroaromatic RNA binders in RIBOTACs yet all converge on the central principle of using organic chemistry to mediate proximity and function. Looking forward, integration of computational modeling, machine learning, and high-throughput chemical synthesis promises to transform degrader discovery into a more predictive and design-driven discipline. This outlook positions organic chemistry not as a supporting detail, but as the driving force that will determine the breadth and impact of proximity-induced therapeutics. From an organic chemistry perspective, the common thread among these modalities is bifunctionality and modular design. We are witnessing a shift from the traditional “one-drug, one-target” model to a “molecular machine” model, where a drug is composed of parts, each part with a specific binding function, and the drug's efficacy comes from orchestrating an interaction between two biological entities. This modular approach puts a premium on linker chemistry, conjugation techniques, and the discovery of small-molecule ligands for challenging biomolecules (E3 pockets, RNA structures, etc.). Synthetic chemistry has enabled the incorporation of unnatural motifs (e.g. phosphonoglycosides for M6P, guanine analogues for AUTAC tags) to mimic or perturb biological signals. It has also delivered elegant solutions to solubility and permeability issues for instance, installing chameleon-like linkers that fold to reduce polarity, or including cleavable masking groups that unveil polar warheads only inside cells. Furthermore, click chemistry and other bioconjugation methods have been indispensable for assembling large constructs like antibody–glycan LYTACs or aptamer conjugates.

As the field progresses, several trends and challenges are apparent. One is selectivity: ensuring that induced-proximity agents discriminate their intended target without hijacking other off-target molecules. This is particularly pertinent for molecular glues (which can have promiscuous effects if they broadly alter an E3's substrate scope) and for RIBOTACs (to avoid global RNA degradation). Rigorous proteomic and transcriptomic profiling, as well as structure-guided tuning of the chemical scaffolds, will be key to refining specificity. Another challenge is resistance – cells may adapt to degraders, for example by mutating the recruited E3 ligase (as seen in patients on CRBN-binding drugs who develop CUL4-CRBN mutations) (Haj-Yahia et al., 2023a). Anticipating this, researchers are exploring dual-degrader strategies (e.g., a PROTAC that can bind two different E3 ligases at once, to reduce reliance on any single ligase) (London, 2024b; Ma et al., 2023), or cycling through different degrader classes to prevent escape.

A very exciting development is the integration of computational design and machine learning in this space. With accumulating data on successful vs. failed PROTACs and glues, algorithms are being trained to predict favorable linker lengths, orientations for ternary complexes, or new E3 ligand pairs. In silico screening for molecular glues, which simulate transient protein–protein contact surfaces, is an emerging discipline melding computational chemistry with structural biology. Likewise, the discovery of RNA-binding small molecules for RIBOTACs is benefitting from AI models trained on known RNA-ligand interactions. On the therapeutic front, induced-proximity drugs could transform treatment paradigms. The ability to degrade rather than inhibit means we can tackle scaffolding proteins and transcription factors that lack enzymatic pockets. It also means potentially overcoming certain drug resistances: for instance, an oncogenic kinase that mutates to resist inhibitors might still be degraded by a PROTAC targeting an invariant surface epitope. Induced-proximity can also have gain-of-function effects e.g., targeted degradation of a disease-causing protein might release feedback inhibition or trigger compensatory pathways beneficially, something a simple inhibitor might not achieve. However, safety will be a focus: complete removal of a protein is a more extreme perturbation than partial inhibition, so ensuring that the targeted protein is truly dispensable (or at least tolerable to lose) is critical. Early clinical data suggests that carefully chosen targets (such as hormone receptors in cancer) can be safely degraded with manageable side effects.

In conclusion, the 2020–2025 period has been one of intense innovation in the realm of targeted degradation and induced proximity. The convergence of synthetic organic chemistry, protein engineering, and cellular biology has yielded an expanded toolkit to control the fate of biomolecules. PROTACs and molecular glues have taught us that “undruggable” was a temporary designation with the right bifunctional molecule, one can drug the undruggable by destroying it. LYTACs and related lysosomal degraders remind us that extracellular targets, once only addressable by antibodies or gene therapies, can now be knocked down by chimera molecules designed at the bench. AUTACs and ATTECs show that even the cell's waste disposal pathways can be redirected at our command. RIBOTACs suggest a future where we might drug the “dark transcriptome” of RNAs involved in disease. This field is inherently interdisciplinary: it requires deep knowledge of organic synthesis to build the complex molecules, structural insight to make them effective, and biological savvy to validate their function. For chemists, it represents a new frontier of molecular design crafting small molecules not just to bind, but to bring together and modulate biological machinery in a deliberate way. The coming years will likely see the first approved targeted degraders, and with them, a validation that induced proximity is not only a powerful laboratory tool but also a viable therapeutic strategy. As our ability to manipulate molecular interactions grows, induced-proximity therapeutics could become a foundational pillar of pharmacology, fulfilling the long-held dream of precision elimination of any pathological factor using a tailor-made molecule. Recent advances in induced-proximity therapeutics have dramatically expanded the chemical biology toolkit for targeted degradation of proteins and RNA. Among these modalities, PROTACs and Molecular Glues have demonstrated robust efficacy in manipulating the proteome, while LYTACs and AUTACs provide complementary lysosomal and autophagic pathways for selective degradation. RIBOTACs further extend the concept to RNA targets, opening new avenues for modulating gene expression. The organic chemistry underlying these strategies linker design, ligand optimization, and bifunctional molecule synthesis remains central to their success, enabling precise control over induced proximity and downstream degradation kinetics. Looking forward, the field faces several challenges and opportunities. Optimizing pharmacokinetics, tissue specificity, and cell permeability will be critical for clinical translation, while expanding the repertoire of ligands and targetable biomolecules promises to broaden therapeutic scope. Integration of computational design, high-throughput screening, and visual mechanistic frameworks can further accelerate rational design of next-generation induced-proximity therapeutics. Collectively, these insights underscore the pivotal role of organic chemistry in shaping future drug discovery and highlight the transformative potential of induced-proximity strategies in oncology, immunology, and neurodegenerative diseases. Ultimately, induced-proximity therapeutics stand at the intersection of biology and organic chemistry. Their feasibility and clinical promise rest not only on mechanistic insight but on the chemist's ability to design, synthesize, and optimize modular molecules that orchestrate precise biological events. Whether through linker engineering, glycan conjugation, guanine tagging, or heteroaromatic RNA binders, the future of this field will be shaped by synthetic creativity. Organic chemistry remains the unifying discipline that transforms conceptual proximity-inducing strategies into tangible therapeutic candidates, underscoring its indispensable role in the continued evolution of targeted degradation.

## CRediT author statement

Mohammad Rizehbandi: Conceptualization, Supervision, Writing – original draft, Writing – review & editing.

Ehsan Dadfar: Data curation, Literature survey, Writing – original draft.

Mahyar Rezaei Nami: Visualization, Figure preparation, Literature survey.

Mahdi Rezaei Nami: Writing – review & editing, Validation.

Mehran Rezaei Nami: Literature survey, Editing and formatting.

## Funding sources

Not applicable.

## Declaration of competing interest

The authors declare that they have no known competing financial interests or personal relationships that could have appeared to influence the work reported in this paper.

## Data Availability

All relevant data supporting the findings of this study are included within the article.
